# Cost‐Effectiveness Analysis of a Maternal Vaccination Program Against Respiratory Syncytial Virus in Norway

**DOI:** 10.1111/irv.70161

**Published:** 2025-09-07

**Authors:** Susanne Gerda Værnø, Francisco Oteiza, Maren Gillebo, Lise Beier Havdal, David Ngaruiya Mwaura, Øyvind Husby, Oddvar Solli, Kristian Lie, Christoffer Bugge

**Affiliations:** ^1^ Oslo Economics Oslo Norway; ^2^ Department of Pediatric and Adolescent Medicine Akershus University Hospital Nordbyhagen Norway; ^3^ Pfizer AS Oslo Norway

**Keywords:** cost‐effectiveness, maternal immunization, maternal vaccination respiratory syncytial virus (RSV), Norway, RSVpreF vaccine

## Abstract

**Background:**

Respiratory syncytial virus (RSV) is recognized as the primary cause of hospitalizations among children with lower respiratory tract infections in developed countries, placing a significant burden on both patients and healthcare systems. The efficacy, safety, and immunogenicity of maternal vaccination with the novel RSVpreF vaccine have been evaluated in a Phase III clinical trial, showing a decreased risk of severe infection in infants. Our study assesses the cost‐effectiveness of the RSVpreF vaccine and seasonal variation of costs in a Norwegian setting.

**Methods:**

A Markov model was used to estimate the clinical outcomes, costs, and quality‐adjusted life years of a hypothetical cohort of Norwegian infants born during a single RSV season. A seasonal vaccination program with RSVpreF vaccine was compared to no intervention by means of an incremental cost‐effectiveness ratio (ICER) from extended healthcare and societal perspectives.

**Results:**

A seasonal maternal vaccination program with RSVpreF in Norway is cost‐effective from both a healthcare and societal perspective, given the Norwegian willingness‐to‐pay threshold range. The program could prevent 27% of the yearly RSV‐associated hospital admissions, as well as 14% and 24% of the yearly RSV‐associated primary care and outpatient visits. A 10% increase/decrease in hospitalization costs during the winter/summer months leads to a 26% reduction in the ICER from a healthcare perspective and turns the intervention into a dominant strategy from a societal one.

**Conclusions:**

Based on the RSVpreF vaccine's list price in Norway, the seasonal vaccination program is cost‐effective from both the healthcare and societal perspectives, considering a willingness‐to‐pay threshold of 500,000 NOK.

## 1 Introduction

Respiratory syncytial virus (RSV) is recognized as the primary cause of hospitalizations among children with lower respiratory tract infections (LRTI) in developed countries [1]. RSV is particularly severe among infants below 1 year of age, making it a leading cause of hospitalizations for this age group [2]. In temperate climates, RSV infections follow a seasonal pattern, with hospitalization rates peaking during winter, coinciding with other respiratory viral infections, and placing a significant burden on patients and healthcare systems [3]. RSV infections in children below 1 year of age in Norway are estimated to contribute to healthcare costs of 30 million NOK each year, production losses of 40 million NOK, and a health loss valued at 11 million NOK [4].

Until recently, the only licensed product in Norway for protecting infants against RSV was the monoclonal antibody palivizumab, which requires monthly injections throughout the RSV season. However, palivizumab is only indicated for infants with high risk of severe disease [5, 6]. Of the approximately 52,000 live births taking place in Norway each year, approximately 360 children have been treated with palivizumab annually since 2015 [7]. Recently, two effective new products have been licensed to protect infants: a maternal vaccine (RSVpreF) and a long‐acting injectable monoclonal antibody (nirsevimab). Nirsevimab has not yet been marketed in Norway, nor has it been assessed for public reimbursement. The efficacy, safety, and immunogenicity of the novel RSVpreF maternal immunization vaccine have been evaluated in a pivotal placebo‐controlled Phase III clinical trial, showing a decreased risk of severe infection [8]. The vaccine is indicated for pregnant women between Weeks 24 and 36 of gestation and provides protection for infants from birth through their first 6 months of life. The vaccine is also indicated for older adults above 18 years of age. The RSVPreF vaccine has been approved for use in Norway, but the current standard of care for newborns is still no treatment.

Cost‐effectiveness analyses of the RSVpreF vaccine have been conducted in Spain, Japan, the United Kingdom, Argentina, the United States, and Canada [9, 10, 11, 12, 13, 14]. The studies from Japan, Spain, the United Kingdom, and Canada find that vaccination is cost‐effective given a willingness‐to‐pay threshold of ¥5 million, €25,000, £20,000, and CAD$50,000, respectively. The analysis from Argentina finds that vaccination is cost‐effective at a willingness‐to‐pay threshold of $10,636 (1 × GDP), provided that the vaccine costs around $75. Similarly, the study from the United States suggests that vaccination can be cost‐effective under specific circumstances, particularly when administered at the ideal gestational and seasonal time. These results are driven by a relatively high incidence of RSV among young children, who are particularly vulnerable to severe RSV disease and whose hospitalizations are significantly resource demanding. However, context‐specific analyses are needed to account for the geographic variation in RSV epidemiology, as well as the significant cross‐country differences in the structure and relative costs for healthcare services.

This study aims to assess the cost‐effectiveness of an RSV prevention strategy (maternal vaccination with the RSVpreF vaccine compared to no vaccination) for infants without high risk of disease in a Norwegian setting. These children, as described above, currently have no other indicated preventive treatment. Assessment of new treatments for introduction into the Norwegian healthcare service must be made against the current standard of care, as per Norwegian guidelines. Further, we investigate how the nature of a seasonal disease like RSV may impact the cost‐effectiveness of a preventive measure like the RSVpreF vaccine when accounting for scenarios with varying costs for hospitalizations during peak winter months and low RSV incidence months and hence characterizing more precisely the pressure on the healthcare system.

## 2 Methods

### 2.1 Model Structure

**FIGURE 1:**
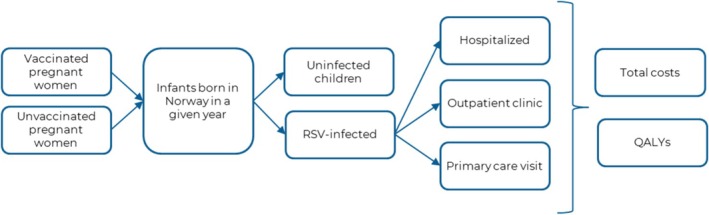
Summary of the health economic model.

A cohort framework and a Markov‐type process were used to depict clinical outcomes and costs related to RSV‐positive respiratory tract infections in newborn infants in Norway (Figure 1) The core model was initially presented in 2023 to the US Centers for Disease Control and Prevention's (CDC) Advisory Committee on Immunization Practices (ACIP) and was updated to include final vaccine efficacy inputs from the MATISSE trial and to reflect the Norwegian setting [8, 15].

The model population comprises 12 monthly rolling birth cohorts, and each cohort is characterized based on their gestational age at birth. Infants were either assumed to be protected against RSV due to maternal vaccination if born to vaccinated mothers, or to have received no intervention and thus be unprotected. Over the course of their first 12 months, infants can become infected with RSV or remain uninfected, based on age‐ and setting‐specific infection and vaccine effectiveness rates. High‐risk, premature children are not included in the patient population.

Only medically attended RSV infections are considered in the model, stratified by care setting into three mutually exclusive groups: hospitalizations, outpatient clinic visits, and primary care visits (i.e., emergency department and general practitioner visits). Each category is characterized by a specific set of costs and health outcomes. We assumed no RSV‐specific mortality and ignored any potential long‐term impacts of RSV infections like recurrent wheezing or asthma. Thus, neither vaccination nor RSV infections have consequences (in terms of costs or health outcomes) beyond those realized in the first 12 months of life.

Expected clinical outcomes are projected for infants in the model population on a monthly basis (i.e., model cycle length = 1 month) based on age, wGA at birth, disease/fatality rates (which may vary by age, wGA at birth, and calendar month), and mother's vaccination status. We estimated the reduction in medically attended RSV cases and the corresponding cost savings and quality‐adjusted life years (QALYs) gained in a maternal vaccination program scenario compared to a scenario with no vaccination. An incremental cost‐effectiveness ratio (ICER) was calculated to assess the cost‐effectiveness of the maternal vaccination program from an extended healthcare sector and a societal perspective, in line with Norwegian guidelines [16]. All costs are expressed in 2024 Norwegian kroner (NOK). Given the 1‐year time horizon, no discounting is applied [17]. A summary of the input parameters included in the model is presented in Table 1.

**TABLE 1:** Model input parameters (base case).

Parameter	Base case input values					Sources
Population						
Pregnant women giving birth (*N*)	51,392					[18]
Livebirths (*N*)	51,980					
Distribution of births by wGA (%)	Full‐term (≥ 37 wGA): 94.1%	Late preterm (32–36 wGA): 5.0%	Early preterm (28–31 wGA): 0.6%	Extreme preterm (≤ 27 wGA): 0.3%		[19]
Births by calendar month (%)	See Table S1A					[18]
RSV epidemiology						
RSV incidence by setting	See Table S2A					[3, 20, 21, 22, 23]
Relative risk of RSV encounters by age group	Full‐term (≥ 37 wGA)	Late preterm (32–36 wGA)	Early preterm (28–31 wGA)	Extreme preterm (≤ 27 wGA)		
0 < 3 months	1.0	1.7	0.5	0.5		[24]
3 < 6 months	1.0	2.5	2.4	2.4		
6 < 12 months	1.0	1.7	6.8	6.8		
Proportion of RSV with LRTI	Hospitalization	Outpatient visit		Primary care visit		
0 < 6 months	100%	65%		65%		[25, 26]
6 < 12 months	100%	50%		30%		
RSV encounters by calendar month (%)	Based on observed distribution of RSV hospitalizations for the 2022/2023 and 2023/2024 seasons. See Table S4A					[27]
All‐cause mortality	See Table S5A					[28, 29]
RSV‐associated mortality	No RSV‐specific mortality					[20]
Intervention						
RSVpreF vaccine uptake	73.8%					[30]
Distribution of pregnant women by fetal wGA at time of vaccination	w32	w33	w34	w35	w36	[31]
	33.5%	24.0%	17.2%	13.8%	11.5%	
Vaccination season	September through January					[30]
Vaccine efficacy, initial (%)^a^	Hospitalization: 84.4%	Outpatient visit: 70.5%		Primary care visit: 70.5%		[32]
Vaccine efficacy, duration	Trial‐based waning over 6 months/linear waning to 0% by 9 months					Assumption
Direct costs						
Vaccine costs	NOK 1997.67					[33]
Treatment costs by setting and age	< 1 months	1 < 2 months	2 < 6 months	6 < 12 months		
Hospitalization—full term (≥ 37 wGA)	NOK 61,358	NOK 58,218	NOK 57,472	NOK 57,196		Table S6A [26, 34, 35, 36]
Hospitalization—preterm (< 37 wGA)	NOK 79,788	NOK 60,468	NOK 59,268	NOK 58,823		
OC visit	NOK 5016					[26]
PC visit	NOK 718					KUHR database
Indirect costs						
Secondary caregivers in workforce (%)	84%					[37]
Average daily wage	NOK 5216					[35]
Hospitalization LOS by age (days)	< 3 months	3 < 6 months		6 < 12 months		
Full term (≥ 37 wGA)	3.2	2.8		2.5		[34]
Preterm (≤ 37 wGA)	5.2	4.4		4.0		[34]
Travel costs, per event	NOK 269					[38]
Utility						
QALY loss due to RSV encounter	Hospitalization	OC visit		PC visit		
Infant	0.0157	0.0061		0.0061		[39]
Caregiver	0.0031	0.0031		0.0031		[40]

Abbreviations: LOS, length of stay; LRTI, lower respiratory tract infection; N, number; QALY, quality‐adjusted life years; RSV, respiratory syncytial virus; URTI, upper respiratory tract infection; wGA, gestational age in weeks.

^a^ Applied only to full term and late pre‐term infants.

### 2.2 Population

The model population includes all liveborn infants in Norway (*n* = 51,980) during a 1‐year period [18]. Liveborn infants were first classified according to their gestational age at birth measured in weeks (wGA), based on Norwegian estimates [19]. Infants were then grouped into four groups: full term (≥ 37 wGA), late preterm (32–36 wGA), early preterm (28–31 wGA), and extreme preterm (≤ 27 wGA) (Table 1). Births were distributed by calendar month according to the average distribution of births in Norway from 2004 to 2023 (Table S1A) [18].

### 2.3 Disease Incidence

The age‐ and setting‐specific RSV infection rates used in the model are presented in Table S2A. RSV hospitalization rates were based on Johannesen et al. [20]. Admission rates were assigned to monthly age categories using the relative admission rates by age reported by Curns et al. [21]. A ratio of one outpatient clinic visit for every two hospitalizations was assumed based on findings from three RSV seasons (2015–2018) in Norway [22]. The incidence of primary care RSV visits for infants up to the age of 5 months was assumed to be five times the incidence of hospitalizations, in line with Li et al. [3]. For children between the ages of 6 and 12 months, we assumed 12.5 primary care visits for each RSV hospitalization, following Cromer et al. [23]. The assumed relative risk of RSV infection by infant's term status at birth and age was based on data from a US study [24]. Yearly incidence rates by setting were assigned to each calendar month based on the observed distribution of RSV hospitalizations in Norway during the first two regular RSV seasons following the Covid‐19 pandemic (2022/2023 and 2023/2024), as reported by the Norwegian Institute of Public Health (Table S3A) [27].

### 2.4 Mortality

All‐cause infant mortality rates by age group were based on estimates of perinatal and infant mortality from Statistics Norway (Table S4A) [28]. Relative risks of mortality by term status at birth were imputed based on estimates from the US CDC, due to lack of evidence from the Norwegian setting [29]. During the nine RSV seasons from 2008 to 2017, a total of eight children below the age of 5 were reported by the Norwegian Cause of Death Registry to have suffered RSV‐related deaths, all of whom had congenital heart disease or other severe comorbidities [3]. Based on these findings, we assumed no RSV‐specific mortality in our model.

### 2.5 Vaccination Strategy and Effectiveness

Maternal vaccination was implemented as a seasonal vaccination program in the base case analysis (September–January), following the recommendations by the US CDC and ACIP [41]. Vaccine uptake was assumed to be 73.8%, based on a 2019 Norwegian study [30]. The distribution of vaccine administration by fetal wGA was based on the observed distribution for maternal Tdap observed between 32 and 36 wGA in a recent US study [42]. An alternative strategy is including the vaccine at the maternity care visit in Week 28, in line with practice in the United Kingdom.

Setting‐specific vaccine effectiveness estimates were derived using the cumulative efficacy data (at 90, 120, 150, and 180 days) for the two primary endpoints in the MATISSE trial [32]. The MATISSE Phase III trial was a global, randomized, double‐blinded, placebo‐controlled study evaluating the efficacy, immunogenicity, and safety of the RSVpreF vaccine in pregnant individuals. A total of 7420 healthy pregnant participants aged ≤ 49 years at 24–36 weeks of gestation were randomized (1:1) to receive either a single 120‐μg dose of RSVpreF or a placebo. The trial's primary efficacy outcomes measured protection against severe and non‐severe RSV‐associated medically attended lower respiratory tract illness (LRTI) in newborns and infants within 180 days of birth.

Efficacy against severe, RSV‐associated medically attended LRTI was used as a proxy for vaccine effectiveness against RSV‐LRTI requiring hospitalization. Efficacy against medically attended RSV‐positive LRTI was used as a proxy for vaccine effectiveness against RSV‐LRTI treated in outpatient clinic or primary care settings. These endpoints were proportionally applied to setting‐ and age‐specific groups using US evidence on the rates at which RSV presents as LRTI, as opposed to upper respiratory tract infection (URTI) [25].

For full‐term and late preterm infants, vaccine effectiveness by age (in months) was derived linearly from the cumulative efficacy datapoints from MATISSE through age 5 to < 6 months. Vaccine effectiveness was assumed to wane linearly to 0% by age 9 to < 10 months (Figure S1A). As the MATISSE study was not statistically powered to provide efficacy estimates among preterm infants, vaccine effectiveness among early and extremely preterm infants, and among infants born < 2 weeks after vaccination, irrespective of term status, was assumed to be 0%.

The MATISSE study found similar adverse event profiles among pregnant women in the RSVpreF vaccine and the placebo cohorts with mostly mild‐to‐moderate reactogenicity [32]. We therefore do not include effects associated with adverse events from vaccination.

### 2.6 Utilities

Health state utility for children without an RSV infection was assumed equal to 1. For children infected with RSV, the utility value employed was dependent on the care setting, regardless of term status at birth. A 14‐day illness period was assumed, irrespective of care setting, leading to QALY losses per RSV episode of 0.157 for hospitalizations and 0.0061 for outpatient clinic and primary care visits, based on time trade‐off estimated from a study of health‐related quality of life weights for RSV‐infected infants [39]. QALY losses for caregivers of infants with RSV, regardless of care setting, were assumed to be 0.0031 based on Global Rating of Health from a recent review [40].

### 2.7 Direct Costs

In Norway, pregnant women are offered a minimum of nine prenatal checkups during which elected vaccines are offered [43, 44]. We assume that the RSVpreF would be included as part of this offering and demand no further healthcare resources. Intervention costs thus consist of vaccine acquisition costs, based on the agreed maximum retail price (excl. VAT) of NOK 1997.76 [33].

The costs of RSV‐related hospitalizations and outpatient clinic visits were calculated based on the corresponding diagnosis‐related group (DRG) codes of each specific encounter, by infant's term status and age at hospitalization (Tables 1 and S5A). Costs in the outpatient setting were based on the relevant DRG code for outpatient consultations regarding infections located in the lower respiratory tract (DRG 904D, NOK 5016) and were assumed identical for all term status groups given a lack of more detailed data.

Costs related to primary care episodes were calculated using data from the Norwegian Control and Payment of Health Reimbursements Database, which includes all emergency department and general practitioner visits in the Norwegian public health system. The average cost of a primary care encounter for respiratory illness among patients under the age of 1 between August 2022 and July 2023 in 2024 terms was NOK 718, applied regardless of age groups and term status (Tables 1 and S6A).

### 2.8 Indirect Costs

In Norway, an infant's main caregiver is entitled to paid maternity leave for between 15 and 42 weeks after birth, when most RSV hospitalizations of infants occur. We thus assume no productivity loss for the main caregivers. However, input from specialists working in pediatric wards informed our assumption that RSV hospitalizations lead to productivity losses for employed secondary caregivers assumed to be equivalent to the hospitalization's length of stay, derived from Wang et al. [34]. We applied the median daily wage of NOK 5216 per day lost based on Norwegian guidelines to a share of caregivers, based on the share of adults aged 30–40 in full‐time employment (84%) [35]. No productivity losses were assumed for caregivers for outpatient clinic or primary care encounters. A cost of NOK 269, reflecting the average cost of transporting an infant to a hospital or local clinic, outpatient clinic, or primary care visits is applied to each encounter, based on estimates from the literature [38].

### 2.9 Sensitivity Analyses

A deterministic sensitivity analysis (DSA) was conducted to determine which variables had the greatest impact on cost‐effectiveness. The DSA was performed by varying disease incidence, vaccine effectiveness, utilities, and costs by ±25%. A probabilistic sensitivity analysis (PSA) was also conducted in which key input parameters were drawn from probability distributions to assess the degree of structural uncertainty of the model. The PSA was performed by Monte Carlo simulation with 1000 iterations (see Table S7A for PSA assumptions).

### 2.10 Alternative Scenarios

Using Norwegian respiratory hospitalization data for 2018–2023, we developed scenarios in which we increased the costs of hospitalizations during the three busiest months of the year (December–February) by 10% increments and reduced these costs during the three least busy months (June–August), up to a 40% increase/decrease [27].

Temperate countries such as Norway have been found to have biennial cycles of RSV activity, with severe seasons followed by less severe seasons [22, 45, 46, 47, 48, 49, 50, 51, 52]. We modeled two scenarios, one in which the incidence of RSV reflects a severe season (similar to 2022/2023) and one in which it reflects a milder season (similar to 2023/2024), based on historical RSV hospitalization rates from Norway. Whereas the 2022/2023 season saw around 10% higher RSV hospitalization rates than the post‐pandemic average, the 2023/2024 season saw around 10% fewer hospitalizations [27].

We included four scenario analyses with different vaccination strategies. First, we included a scenario in which vaccination takes place only during three (instead of five) calendar months (November–January), targeting infants born during the three calendar months with the highest average incidence of RSV hospitalizations in Norway since 2022 (December–February). This reflects a hypothetical scenario in which Norwegian health authorities have perfect information and can tailor each vaccination calendar in advance to align precisely with RSV incidence rates. We also included two additional scenarios that better reflect the uncertainty that health authorities operate under: a 7‐month vaccination strategy (August–February) and a year‐round vaccination strategy.

Our base case scenario assumes production losses for employed second caregivers of the sick child equal to the RSV hospitalization's LOS. In a scenario analysis, we also included the cost of unpaid time lost by caregivers, in line with recommendations from the health economics literature [53, 54]. Lost unpaid time to disease by primary caregivers and out of work secondary caregivers is modeled by assigning a non‐market average daily wage of NOK 2759 based on Norwegian guidelines [35] and is assumed equivalent to the length of stay of the infant's hospitalization. No production losses are assumed for outpatient clinic or primary care visits.

We explore the impact of discounting the base case price of the vaccine (NOK 1997.76) by 10% increments, up to a 30% discount level.

## 3 Results

### 3.1 Base Case Analysis

The key outcomes and costs involved in each of the two different strategies compared in the base case analysis are shown in Table 2 Out of the 51,980 assumed live births in Norway each calendar year, the seasonal maternal vaccination strategy with vaccination of pregnant women with expected delivery from September through January would result in 14,950 infants being born to vaccinated mothers. A seasonal maternal vaccination program against RSV in Norway could prevent 27% of the yearly RSV‐associated hospital admissions (*n* = 385), 14% of primary care visits (*n* = 833), and 24% of outpatient visits (*n* = 93). This leads to 16 additional QALYs for infants and their caregivers, compared to no vaccination.

**TABLE 2:** Base case analysis results.

	Maternal vaccination	No intervention	Difference
Vaccination program (whole year)			
Pregnant women	51,392	51,392	0
Live births	51,980	51,980	0
Live births to vaccinated mothers	14,950	0	14,950
Healthcare resource use			
Hospitalizations	1040	1425	−385
Primary care visits	5053	5886	−833
Outpatient visits	299	392	−93
HRQoL			
QALYs infants	51,602.55	51,590.86	11.69
QALYs caregivers lost	19.82	23.88	−4.06
Incremental QALYs			15.75
Costs (NOK)			
Hospitalizations	60,640,865	83,070,712	−22,429,847
Primary care visits	3,628,699	4,226,623	−597,924
Outpatient visits	1,500,181	1,967,793	−467,611
Vaccination program	29,528,087	0	29,528,087
Travel costs	1,720,139	2,072,816	−352,676
Total costs (extended healthcare perspective)	97,017,972	91,337,944	5,680,028
Production losses	15,577,866	21,067,938	−5,490,072
Total costs (societal perspective)	112,595,838	112,405,882	189,956
ICER—cost per QALY			
Healthcare perspective			360,625
Societal perspective			12,060

*Note:* Numbers are rounded in the table, resulting in a minor discrepancy in ICER reported and the number reported.

Abbreviations: HRQoL, health‐related quality of life; ICER, incremental cost‐effectiveness ratio; QALY, quality‐adjusted life years.

From an extended healthcare sector perspective, the maternal vaccination program against RSV costing approximately NOK 30 million led to a net cost of NOK 6 million versus no intervention, the difference mainly due to the reduction in costs for RSV‐associated hospitalizations under the vaccination scenario (NOK 22 million). This results in an ICER of NOK 360,625. The reduction in hospitalizations significantly reduces production losses leading to savings of approximately NOK 5.5 million per season. The resulting ICER from a societal perspective is NOK 12,060.

### 3.2 Sensitivity Analyses

The results of the DSA highlight four variables as key for the vaccination program's cost‐effectiveness (Figure S2A). The cost and efficacy of the RSVpreF vaccine are two of them, whereas the incidence of RSV‐related hospitalizations, as well as their cost, also have a significant impact on the estimations. The PSA shows that all model iterations imply health gains, whereas a significant share of them additionally leads to cost savings under both the healthcare and societal perspectives (Figure S3A). The PSA generates an ICER from an extended healthcare perspective below 500,000 NOK in 55% of all iterations.

### 3.3 Alternative Scenarios

A 10% increase/decrease in hospitalization costs during the winter/summer months leads to a 28% reduction in ICER (NOK 279,344) from a healthcare perspective and turns the intervention into a dominant strategy from a societal perspective (Table 3 and Figure S4A). When hospitalization costs change by 40%, the intervention becomes dominant also from a healthcare perspective. RSV season severity and vaccination strategy both have significant impacts on the ICER. A year‐round vaccination strategy is estimated to avoid up to 41% of yearly RSV hospitalizations (Table S8A) but increases the ICER significantly. The inclusion of lost unpaid time to disease for hospitalized infants' caregivers makes the intervention dominant from a societal perspective. The intervention becomes dominant from a healthcare perspective at a 20% discounted price.

**TABLE 3:** Results from alternative scenario analyses.

Base case ICER	HC‐P: NOK 360625		ICER (NOK)	
	SOC‐P: NOK 12060			
Parameter	Base case value	Scenario value	HC‐P	SOC‐P
Seasonal hospitalization costs	No variation in costs across months	10% higher/lower	252,041	Dominant
		20% higher/lower	143,457	Dominant
		30% higher/lower	34,873	Dominant
		40% higher/lower	Dominant	Dominant
Severity of RSV season	Average	Mild	567,024	218,458
		Severe	191,472	Dominant
Vaccination strategy	Seasonal (Sep–Jan)	3 months (Nov–Jan)	99,452	Dominant
		7 months (Aug–Feb)	642,996	298,545
		Whole year	1,711,421	1,373,187
Production losses	Workdays lost by one caregiver, equal to hospitalization LOS	Add value of lost free time for first caregiver (and second if not employed) equal to hospitalization LOS	360,625	Dominant
Vaccine price	NOK 1997.76	10% discount	173,151	Dominant
		20% discount	Dominant	Dominant
		30% discount	Dominant	Dominant
Vaccination strategy	Women vaccinated between Weeks 32 and 36	Vaccination at Week 28 (1/3 per Weeks 27–29)	295,316	Dominant

Abbreviations: HC‐P, healthcare perspective; ICER, incremental cost‐effectiveness ratio; LOS, length of stay; SOC‐P, societal perspective.

## 4 Discussion

Our study suggests that a seasonal maternal vaccination program against RSV with the RSVpreF vaccine could prevent RSV‐associated hospital admissions in infants, as well as primary care and outpatient clinic visits. Based on the RSVpreF vaccine's list price in Norway, the seasonal vaccination program is cost‐effective from both the healthcare and societal perspectives considering a willingness to pay threshold of 500,000 NOK.

Our findings are in line with recently published cost‐effectiveness studies for maternal vaccination with the RSVpreF vaccine conducted in other countries. A Spanish study found that year‐round vaccination is a dominant strategy compared to no intervention [9], driven by significantly higher incidence rates for RSV‐associated hospitalizations and outpatient visits than what has been documented in Norway [55]. A study from Japan found that year‐round RSVpreF vaccination of pregnant women combined with their current practice of palivizumab treatment for premature and high‐risk infants was cost‐effective [10]. An analysis of six European countries found that year‐round maternal vaccination with the RSVpreF vaccine was dominant from a healthcare perspective in Finland and cost‐effective in Scotland, compared to no intervention [56]. A cost‐effectiveness analysis from Norway found that year‐round maternal vaccination with RSVpreF was not cost‐effective when compared to seasonal monoclonal antibody programs, but this study assumed a significantly lower price for nirsevimab and was conducted before RSVpreF efficacy results from the MATISSE trial had been published [3]. Our study contributes to this body of evidence with an analysis from the Norwegian context based on published clinical study results, Norwegian epidemiological studies, and health registry data.

Our analysis shows that a seasonal vaccination program is more cost‐effective than year‐round vaccination. However, in practice, the cost‐effectiveness of the seasonal program relies on how well vaccination timing aligns with the RSV season and the severity of the season. Taking into account practical implementation aspects when considering such a program will be important, particularly in ensuring that vaccination efforts are well timed and integrated into existing healthcare services. Reliable surveillance data will play a key role in monitoring RSV trends and guiding decisions on the timing of vaccination. This study includes several assumptions that may underestimate the true cost‐effectiveness of a vaccination program. In line with Norwegian evidence, we assume no preventable RSV‐specific mortality [20]. The inclusion of mortality would significantly impact health outcomes and productivity losses [9, 10]. Further, studies suggest that RSV infections during infancy may have long‐term effects, including increased risk of wheezing and asthma during childhood, which we have not included [57, 58]. Vaccine effectiveness was modeled based only on the MATISSE trial's two primary endpoints, both defined in terms of medically attended RSV‐associated LRTI. Secondary and exploratory endpoints from the trial suggest that RSVpreF may also provide a degree of protection against RSV‐associated URTI, not included in this analysis [8]. Finally, no protection against RSV was assumed for early and extremely preterm infants (≤ 31 wGA), although preliminary findings from an observational seroepidemiology study of naturally acquired RSV antibody study suggest a degree of transplacental transfer of maternal RSV antibodies for this group [59].

Further, this study has three important limitations. The first is the potential benefits to vaccinated pregnant women, as we only account for a reduction in RSV infections among infants. Similarly, potential side effects on the vaccinated women are not included. The second relates to the impact reduced pressure on hospital capacity could have on other patients in need of care. Finally, evidence from Norway suggests a degree of interference between respiratory illnesses such as rhinovirus and influenza [60, 61]. However, the impact of vaccination against RSV in a small share of the population would have on the epidemiology of these diseases is unclear. There are significant differences between countries in terms of factors such as epidemiology, healthcare costs, and vaccine coverage, impacting the cost effectiveness of a maternal immunization program. For this reason, our findings have limited generalizability to countries different from Norway.

## 5 Conclusion

This study suggests that seasonal maternal vaccination with the RSVpreF could avoid a high number of hospitalizations, outpatient clinic visits, and primary care visits, at a reasonable cost. The cost‐effectiveness of a vaccination program is sensitive to the timing, intensity, and duration of each RSV season, as well as the marginal costs of hospitalizations during peak respiratory illness season. Continued RSV surveillance and marginal hospitalization cost data are essential. Based on the RSVpreF vaccine's list price in Norway, the seasonal vaccination program is cost‐effective from both the healthcare and societal perspectives considering a willingness‐to‐pay threshold of 500,000 NOK.

## Author Contributions

**Susanne Gerda Værnø:** investigation, writing – original draft, methodology, formal analysis, visualization, data curation. **Francisco Oteiza:** investigation, writing – original draft, methodology, formal analysis, project administration, validation. **Maren Gillebo:** investigation, writing – original draft, methodology, formal analysis, visualization, data curation. **Lise Beier Havdal:** writing – review and editing, methodology, conceptualization, writing – original draft. **David Ngaruiya Mwaura:** writing – review and editing, writing – original draft, conceptualization, methodology, project administration. **Øyvind Husby:** writing – review and editing, writing – original draft, conceptualization, methodology. **Oddvar Solli:** writing – review and editing, writing – original draft, conceptualization, methodology. **Kristian Lie:** writing – review and editing, methodology, conceptualization, writing – original draft. **Christoffer Bugge:** investigation, writing – original draft, writing – review and editing, formal analysis, methodology, funding acquisition, project administration, supervision.

## Ethics Statement

This study was based on previously conducted studies and does not contain any new studies with human participants or animals performed by any of the authors and thus did not require ethical approval.

## Conflicts of Interest

Susanne Gerda Værnø, Francisco Oteiza, Maren Gillebo, and Christoffer Bugge are employed by Oslo Economics AS. Oslo Economics conducts research and consulting work for public and private healthcare providers as well as pharmaceutical companies. David Ngaruiya Mwaura, Øyvind Husby, Oddvar Solli, and Kristian Lie are employees of Pfizer AS and own shares in Pfizer Inc. Lise Beier Havdal declares no conflicts of interest.

## Peer Review

The peer review history for this article is available at https://www.webofscience.com/api/gateway/wos/peer‐review/10.1111/irv.70161.

## Supporting information


**Appendix S1:** Supporting Information.

## Data Availability

Data sharing is not applicable to this article as no new data was created or analyzed in this study.
