# Supplementary material for: Cost‐Effectiveness Analysis of a Maternal Vaccination Program Against Respiratory Syncytial Virus in Norway
Source: Influenza Other Respir Viruses. 2025 Sep 7;19(9):e70161. doi: 10.1111/irv.70161 (PMC12414795; doi:10.1111/irv.70161)
Supplement: Supplementary file 1 — Appendix S1: Supporting Information. [file IRV-19-e70161-s001.docx]

###### Supplementary Appendix

“Cost‑Effectiveness Analysis of a Maternal Immunization Campaign against Respiratory Syncytial Virus in Norway”

## Additional figures and tables

Table SA 1: Distribution of live births by calendar month in Norway, average 2004-2023

| Month | Share of births | Month | Share of births | Month | Share of births |
| --- | --- | --- | --- | --- | --- |
| January | 8,5% | May | 9,0% | September | 8,7% |
| February | 7,8% | June | 8,7% | October | 8,3% |
| March | 8,4% | July | 9,3% | November | 7,3% |
| April | 8,4% | August | 9,0% | December | 6,7% |

Source: Statistics Norway [1].

Table SA 2: RSV-specific yearly incidence rates (per 1 000 person years)

| Age in month | Primary care visits | Outpatient visits | Hospitalizations |
| --- | --- | --- | --- |
| <1 | 86,71 | 18,52 | 37,31 |
| 1-<2 | 157,06 | 32,64 | 65,77 |
| 2-<3 | 221,87 | 23,42 | 47,22 |
| 3-<4 | 201,49 | 19,55 | 39,41 |
| 4-<5 | 246,16 | 17,06 | 34,39 |
| 5-<6 | 345,21 | 13,70 | 27,61 |
| 6-<7 | 231,54 | 9,19 | 18,52 |
| 7-<8 | 192,95 | 7,66 | 15,44 |
| 8-<9 | 178,48 | 7,09 | 14,28 |
| 9-<10 | 202,60 | 8,04 | 16,21 |
| 10-<11 | 144,71 | 5,75 | 11,58 |
| 11-<12 | 144,71 | 5,75 | 11,58 |

Source: RSV-hospitalization rates estimated based on Johannesen et al (2022) [3]. Admission rates were assigned to monthly age categories using the relative admission rates by age from Curns et al (2024) [4]. Outpatient consultations based on hospitalization rates and relative number of hospitalizations vs outpatient visits found by Havdal et al (2022) [5]. Primary care contacts based on estimates from Li et al (2022) and Cromer et al (2014) [6, 7].

Table SA 3: Share of RSV-hospitalizations by calendar month

| Month | Share of hospitalizations | Month | Share of hospitalizations | Month | Share of hospitalizations |
| --- | --- | --- | --- | --- | --- |
| January | 34,9% | May | 0,5% | September | 0,3% |
| February | 22,2% | June | 0,1% | October | 2,0% |
| March | 10,6% | July | 0,1% | November | 5,5% |
| April | 4,0% | August | 0,2% | December | 19,6% |

Source: based on the observed distribution of RSV-hospitalizations in Norway over the course of the 2022/2023 and 2023/2024 seasons, provided by the Norwegian Institute of Public Health [11].

Table SA 4: Infant (all-cause) mortality rate (per 1 000 live births), by age

| Age in month | Full-term (≥37 wGA) | Late preterm (32-36 wGA) | Early Preterm (28-31 wGA) | Extreme preterm (≤27 wGA) |
| --- | --- | --- | --- | --- |
| <1 | 0,43 | 3,29 | 18,16 | 205,13 |
| 1-<2 | 0,91 | 3,08 | 8,85 | 36,83 |
| 2-<3 | 0,91 | 3,08 | 8,85 | 36,83 |
| 3-<4 | 0,91 | 3,08 | 8,85 | 36,83 |
| 4-<5 | 0,91 | 3,08 | 8,85 | 36,83 |
| 5-<6 | 0,91 | 3,08 | 8,85 | 36,83 |
| 6-<7 | 0,60 | 1,54 | 4,41 | 18,00 |
| 7-<8 | 0,60 | 1,54 | 4,41 | 18,00 |
| 8-<9 | 0,60 | 1,54 | 4,41 | 18,00 |
| 9-<10 | 0,60 | 1,54 | 4,41 | 18,00 |
| 10-<11 | 0,60 | 1,54 | 4,41 | 18,00 |
| 11-<12 | 0,60 | 1,54 | 4,41 | 18,00 |

Source: For infants 0<1 month old the estimated morality rate for children under 4 weeks old from Statistics Norway was applied [12]. For infants between 1 and 12 months old, Statistics Norway presents a single rate which we applied as a linearly decreasing function over age [12]. Relative risks of mortality by term status at birth were imputed based on estimates from the US CDC, due to lack of evidence from the Norwegian setting [13].

Table SA 5: Hospitalization costs (NOK, 2024)

|  | Subgroup of interest | | | |  |
| --- | --- | --- | --- | --- | --- |
| **Cost item** | **Full term**  **(≥ 37 wGA)** | **Late preterm**  **(32–36 wGA)** | **Early preterm**  **(28–31 wGA)** | **Ext. preterm**  **(≤ 27 wGA)** | **References** |
| Hospitalization costs, non-ICU (per episode, by age) | | | | | |
| <1 months | 55 905 | 55 905 | 55 905 | 55 905 | DRG 390 [9] |
| 1 to <2 months | 55 435 | 55 435 | 55 435 | 55 435 | DRG 98A [9] |
| 2 to <6 months | 55 435 | 55 435 | 55 435 | 55 435 | DRG 98A [9] |
| 6 to <12 months | 55 435 | 55 435 | 55 435 | 55 435 | DRG 98A [9] |
| Hospitalization costs, ICU (per episode, by age) | | | | | |
| <1 months | 162 439 | 522 532 | 522 532 | 522 532 | DRG 389C, 388C [9] |
| 1 to <2 months | 109 813 | 153 775 | 153 775 | 153 775 | One day of ICU ward, rest general ward [19, 20] |
| 2 to <6 months | 95 236 | 130 324 | 130 324 | 130 324 |  |
| 6 to <12 months | 89 830 | 121 627 | 121 627 | 121 627 |  |
| Hospitalization costs, average (per visit, by age) | | | | | |
| <1 months | 61 358 | 79 788 | 79 788 | 79 788 | Weighted average ICU, non-ICU [18] |
| 1 to <2 months | 58 218 | 60 468 | 60 468 | 60 468 |  |
| 2 to <6 months | 57 472 | 59 268 | 59 268 | 59 268 |  |
| 6 to <12 months | 57 196 | 58 823 | 58 823 | 58 823 |  |

Notes: ICU: intensive care unit; wGA: weeks of gestational age at birth; DRG: diagnosis-related group. The costs of hospitalizations in the general ward were based on two specific DRG codes, one for infants under the age of one month (DRG 390), and another for older infants (DRG 98A). These costs were applied regardless of term status at birth. ICU hospitalizations for infants under the age of one month were assigned DRG codes describing the costs associated with serious respiratory illness among newborns (DRG 388C and 389C). For infants aged two months and older, we assumed that those in need of intensive care spend one day at the ICU, and that the rest of their admission is spent in the general pediatric ward. Standard rates for both ICU and general ward stays per day were then applied for this group, using estimates from the Norwegian Medicines Agency [19]. The length of RSV hospitalizations for infants in need of intensive care was based on the Norwegian estimates from a recent study of RSV hospitalizations in seven European countries [20]. Based on a recent study of RSV incidence in Iceland, we assume that 5,12% of infants hospitalized due to RSV require intensive care unit (ICU) services [18]. Overall hospitalization costs are therefore a weighted average of the costs associated with hospitalizations requiring ICU, and those of a regular hospitalization in the pediatric ward. DRG codes are assigned weights representing the average total cost of each encounter in terms of a general unit cost set at NOK 52 248 for 2024 [19]

Figure SA 1: Vaccine effectiveness against RSV-LRTI requiring hospitalization and RSV-LRTI treated in PC or OC

|  |
| --- |

Notes: Efficacy is assumed to be 0% for early and extreme preterm infants, as well as for those vaccinated up to two weeks before birth. RSV-H RSV lower respiratory tract infection requiring hospitalization, RSV-PC/OC: medically attended RSV lower respiratory tract infection treated at outpatient clinic or primary care settings.

Table SA 6: ICPC-2 diagnostic codes included in respiratory illness encounters with the Norwegian Primary Healthcare sector (KHUR)

| ICPC-2 codes |
| --- |
| R01, R02, R03, R04, R05, R06, R07, R08, R09, R21, R24, R25, R27, R29, R33, R72, R74, R75, R76, R77, R78, R79, R80, R81, R82, R83, R99, R991, R992. |

Note: ICPC-2, International Classification of Primary Care, version 2.

Table SA 7: Parameters included in the probabilistic sensitivity analysis

|  | Distribution | Parameters |
| --- | --- | --- |
| Disease incidence |  |  |
| RSV hospitalization | Normal | Mean: 28  SE: 3 |
| RSV primary care visits | Normal | Mean: 206  SE: 21 |
| RSV outpatient visits | Normal | Mean: 14  SE: 1 |
| Effectiveness of maternal vaccination | Beta | Alpha: 18.02:  Beta:4.02 |
| Cost of maternal vaccination | Normal | Mean: 1,997.76  SE: 199.78 |
| Direct costs of disease |  |  |
| RSV hospitalization | Normal | Mean: 58,310.29  SE: 5,831.03 |
| RSV primary care visits | Normal | Mean: 718.11  SE: 71.81 |
| RSV outpatient visits | Normal | Mean: 5,015.81  SE: 501.58 |
| Disutility |  |  |
| RSV hospitalization | Normal | Mean: 5.74  SE: 359.26 |
| RSV primary care visits | Normal | Mean: 2.24  SE: 362.76 |
| RSV outpatient visits | Normal | Mean: 2.24  SE: 362.76 |

Figure SA-2: Tornado diagram DSA results (healthcare perspective)

|  |
| --- |
|  |

Note: Figure shows the parameters with the largest impact on the ICER (healthcare perspective) compared to the base case analysis resulting from the DSA.

Figure SA-3: Scatterplot of results for the PSA

| Healthcare perspective |
| --- |
|  |
| **Societal perspective** |
|  |

Figure SA-4: ICER curve for scenarios of seasonal hospitalization costs (NOK/QALY)

|  |
| --- |
|  |

Note: winter months defined as December through February, summer months defined as June to August. 0% increase/decrease represents base case analysis results.

Table SA-8: Results based on alternative scenario with year-round vaccination

|  | Maternal Immunization | No intervention | Difference |
| --- | --- | --- | --- |
| **Vaccination campaign (number)** |  |  |  |
| Pregnant women (whole year) | 51 392 | 51 392 | 0 |
| Live births (whole year) | 51 980 | 51 980 | 0 |
| Live births to vaccinated mothers | 37 727 |  | 37 727 |
| % of infants adequately protected | 97,6% |  |  |
| **Healthcare resource use (number)** |  |  |  |
| Hospitalizations | 871 | 1425 | -554 |
| Primary care visits | 4 603 | 5 886 | -1 283 |
| Outpatient visits | 264 | 392 | -129 |
| **HRQoL (in QALYs)** |  |  |  |
| QALYs infants | 51608,16 | 51590,86 | 17,30 |
| QALYs caregivers lost | 17,79 | 23,88 | -6,09 |
| Incremental QALYs |  |  | 23,39 |
| **Costs (million NOK)** |  |  |  |
| Hospitalizations | 50688162 | 83070712 | -32382550 |
| Primary care visits | 3305150 | 4226623 | -921473 |
| Outpatient visits | 1322565 | 1967793 | -645228 |
| Vaccination program | 74516752 |  | 74516752 |
| Travel costs | 1543940 | 2072816 | -528876 |
| Total costs (healthcare perspective) | 131376568 | 91337944 | 40038624 |
| Production losses | 13154959 | 21067936 | -7912979 |
| Total costs (societal perspective) | 144531528 | 112405882 | 32125646 |
|  |  |  |  |
| **ICER – Cost per QALY** |  |  |  |
| Healthcare perspective |  |  | 1711421 |
| Societal perspective |  |  | 1373187 |

# References

1. Statistics Norway. *05531: Live births, by month 1966 - 2023 [Accessed 29-08-2024]*. Available from: <https://www.ssb.no/en/statbank/table/05531>.

2. Norman, M., et al., *Preterm birth in the Nordic countries-Capacity, management and outcome in neonatal care.* Acta Paediatr, 2023. **112**(7): p. 1422-1433.

3. Johannesen, C.K., et al., *Age-Specific Estimates of Respiratory Syncytial Virus-Associated Hospitalizations in 6 European Countries: A Time Series Analysis.* J Infect Dis, 2022. **226**(Suppl 1): p. S29-S37.

4. Curns, A.T., et al., *Respiratory Syncytial Virus-Associated Hospitalizations Among Children <5 Years Old: 2016 to 2020.* Pediatrics, 2024. **153**(3).

5. Havdal, L.B., et al., *The burden of respiratory syncytial virus in children under 5 years of age in Norway.* Journal of Infection, 2022. **84**(2): p. 205-215.

6. Li, X., et al., *Cost-effectiveness of Respiratory Syncytial Virus Disease Prevention Strategies: Maternal Vaccine Versus Seasonal or Year-Round Monoclonal Antibody Program in Norwegian Children.* J Infect Dis, 2022. **226**(Suppl 1): p. S95-s101.

7. Cromer, D., et al., *The burden of influenza in England by age and clinical risk group: a statistical analysis to inform vaccine policy.* J Infect, 2014. **68**(4): p. 363-71.

8. Rha, B., et al., *Respiratory Syncytial Virus-Associated Hospitalizations Among Young Children: 2015-2016.* Pediatrics, 2020. **146**(1).

9. Helsedirektoratet. *Innsatsstyrt finansiering (ISF) – regelverk [Accessed 29/08/2024]*. 2024; Available from: <https://www.helsedirektoratet.no/tema/finansiering/innsatsstyrt-finansiering-og-drg-systemet/innsatsstyrt-finansiering-isf>.

10. Rainisch, G., et al., *Estimating the impact of multiple immunization products on medically-attended respiratory syncytial virus (RSV) infections in infants.* Vaccine, 2020. **38**(2): p. 251-257.

11. FHI. *Ukerapporter om covid-19, influensa og andre luftveisinfeksjoner*. 2024; Available from: <https://www.fhi.no/publ/statusrapporter/luftveisinfeksjoner/>.

12. Statistics Norway. *08373: Perinatal and infant mortality, by contents and year [Accessed 29-08-2024]*. Available from: <https://www.ssb.no/en/statbank/table/08373>.

13. National Vital Statistics System, *Mortality 1999-2020 on CDC Wonder Online Database*. 2021, Centers for Disease Control and Prevention.

14. Hansen, B.T., et al., *Predictors of maternal pertussis vaccination acceptance among pregnant women in Norway.* Hum Vaccin Immunother, 2024. **20**(1): p. 2361499.

15. Law, A.W., et al., *106. High Maternal Tdap Vaccine Uptake During Early Part of Vaccination Window: Implications for Future Maternal Vaccines.* Open Forum Infectious Diseases, 2022. **9**(Supplement_2).

16. Kampmann, B., et al., *Bivalent Prefusion F Vaccine in Pregnancy to Prevent RSV Illness in Infants.* N Engl J Med, 2023. **388**(16): p. 1451-1464.

17. Felleskatalogen. *Abrysvo [Accessed 29/08/2024]*. 2024; Available from: <https://www.felleskatalogen.no/medisin/abrysvo-pfizer-734400>.

18. Oskarsson, Y., et al., *Clinical and Socioeconomic Burden of Respiratory Syncytial Virus in Iceland.* Pediatr Infect Dis J, 2022. **41**(10): p. 800-805.

19. DMP. *Enhetskostnadsdatabase [Accessed: 29/08/2024]*. 2024; Available from: <https://www.dmp.no/offentlig-finansiering/metodevurdering-av-medisinske-produkter/legemidler/innsending-av-dokumentasjon/enhetskostnadsdatabase>.

20. Wang, X., et al., *Respiratory Syncytial Virus-Associated Hospital Admissions and Bed Days in Children <5 Years of Age in 7 European Countries.* J Infect Dis, 2022. **226**(Suppl 1): p. S22-s28.

21. Statistics Norway. *13564: Sysselsatte (1 000 personer), etter alder, yrkesstatus, vanlig arbeidstid (heltid/deltid), statistikkvariabel, år og kjønn [Accessed 29-08-2024]*. Available from: <https://www.ssb.no/statbank/table/13564/tableViewLayout1/>.

22. Moger, T.A. and I.S. Kristiansen, *Direct and indirect costs of the Norwegian breast cancer screening program*. 2012, University of Oslo, Health Economics Research Programme.

23. Roy, L.M.C., *Deriving health utility weights for infants with Respiratory Syncytial Virus (RSV)*. 2013.

24. Glaser, E.L., et al., *Impact of Respiratory Syncytial Virus on Child, Caregiver, and Family Quality of Life in the United States: Systematic Literature Review and Analysis.* J Infect Dis, 2022. **226**(Suppl 2): p. S236-s245.
